# Dr. Charles C. Deming

**Published:** 1917-05

**Authors:** 


**Dr. Charles C. Deming,** N. Y. Univ. 1869, died at his home in Friendship, N. Y., March 5, aged about 65. He was an ex-president of the Allegany Co. Medical Society, formerly conducted a sanatorium and was for a time health officer of the town.

